# Development of a Smart Splint to Monitor Different Parameters during the Treatment Process

**DOI:** 10.3390/s20154207

**Published:** 2020-07-29

**Authors:** José María De Agustín Del Burgo, Fernando Blaya Haro, Roberto D’Amato, Juan Antonio Juanes Méndez

**Affiliations:** 1 Campus Miguel de Unamuno, Universidad de Salamanca, 37007 Salamanca, Spain; id00792219@usal.es (J.M.D.A.D.B.); jajm@usal.es (J.A.J.M.); 2 ETSIDI-Departamento de Ingeniería Mecánica, Química y Diseño Industrial, Universidad Politécnica de Madrid (UPM), Ronda de Valencia 3, 28012 Madrid, Spain; fernando.blaya@upm.es

**Keywords:** smart splint, biomedical sensor, iot, additive manufacturing, personalized medicine, health monitoring

## Abstract

For certain musculoskeletal complex rupture injuries, the only treatment available is the use of immobilization splints. This type of treatment usually causes discomfort and certain setbacks in patients. In addition, other complications are usually generated at the vascular, muscular, or articular level. Currently, there is a really possible alternative that would solve these problems and even allows a faster and better recovery. This is possible thanks to the application of engineering on additive manufacturing techniques and the use of biocompatible materials available in the market. This study proposes the use of these materials and techniques, including sensor integration inside the splints. The main parameters considered to be studied are pressure, humidity, and temperature. These aspects are combined and analyzed to determine any kind of unexpected evolution of the treatment. This way, it will be possible to monitor some signals that would be studied to detect problems that are associated to the very initial stage of the treatment. The goal of this study is to generate a smart splint by using biomaterials and engineering techniques based on the advanced manufacturing and sensor system, for clinical purposes. The results show that the prototype of the smart splint allows to get data when it is placed over the arm of a patient. Two temperatures are read during the treatment: in contact with the skin and between skin and splint. The humidity variations due to sweat inside the splint are also read by a humidity sensor. A pressure sensor detects slight changes of pressure inside the splint. In addition, an infrared sensor has been included as a presence detector.

## 1. Introduction

The new industry is experiencing an enormous growth, thanks to the use of techniques that include advanced manufacturing and industrial design. Moreover, the optimization of the process [1,2,3], the development of new materials by manufacturers [4,5,6], or the decrease of environmental costs [7] are some achievements that make it possible to expand these technics further than the industrial area. Nowadays, these techniques are being transferred to medicine areas, by applications known as bioengineering [8].

Among all emerging technologies in the area of bioengineering, the implementation of additive manufacturing (AM) in the healthcare industry has led to several benefits. The design freedom allows customization of surgical equipment, medical devices, and implants. In fact, anatomy for pedagogical objectives [9,10], craniomaxillofacial surgery [11], oral appliance therapy for snoring and obstructive sleep apnea (OSA) [12,13], orthopedics surgery [14], and rehabilitation [15] are some examples in which the benefits of fused filament fabrication (FFF) technology could improve the clinical course of patients. These new techniques allow to improve wearing comfort and better hygiene due to the bio-compatible and sterilizable properties [16]. Over this fields, many developments have been carried out in recent years, like exoskeletons [17], prosthesis, or assistive devices. These specialties are in demand in developed countries [18], but a level of maturity and technological development is necessary to promote a change in the actual model. Moreover, both orthopedics and rehabilitation are specialties of medicine very conducive and receptive to all kinds of technological developments or applications in the field of research, development, and innovation (R&D&I).

Thanks to the use of different methods of industrial three-dimensional digitization and reverse engineering, advanced manufacturing has generated a recent emergence of solutions. Based on additive manufacturing it has allowed a great advance in products of mass use such as splints, braces, prostheses, or assistive devices [19]. Moreover, these techniques, combined with new sensors and actuators, have revolutionized the applications of exoskeletons by the hand of robotics [20]. Products such as splints made by additive manufacturing offer great advantages over classical methods of permanent and splinting (splinting) or commercial immobilization (braces, corsets, etc.). Some of these advantages are adaptation, personalization, hygiene, materials used, or environmental impact, among others. The incursion of these new 3D techniques in medical processes allows to include smart systems to analyze different states of the medical evolution process [21]. This study proposes to combine 3D techniques with electronic systems to develop a new era of splints that have not been achieved before. The use of these kind of systems, which include small electronic boards, programming systems, and data communication, would make possible to monitor parameters as temperature and pressure. These parameters would be sent to an application running on a portable device and compared in a database with other similar treatments data or sent to the doctor to see if the process is evolving correctly. This way, it is possible to include the smart splints into the use of big data in healthcare and the Internet of Things (IoT) movement [22,23]. In fact, it has been shown that the use of the IoT, allows data exchange through network connectivity and devices integrated with hardware, software, and sensors [24]. This, applied to medicine, will lead to a reduction in costs and inefficiencies and better patient satisfaction in terms of medical care. One of the factors for the use of IoT in medicine and in rehabilitation [25] are medical devices (things) integrated with a detection technology for the continuous acquisition of physiological data from patients. For these reasons, the purpose of this study is to present how to design and manufacture a smart and functional splint in order to monitor different physiological parameters of the patient and of the device (skin temperature, humidity, pressure, and color changes on the skin). In this manner, it is possible to have a continuous data acquisition that allows to improve the treatment outcomes and to reduce recovery times and healthcare costs.

## 2. Material and Methods

In this section, it will be explained and presented all the steps followed in this study in order to 3D model, design, prototype manufacture, and assemble a smart and functional arm splint. The arm of a 29-year-old volunteer as a model was used. In this case, there is no lesion, but it will be used as the basis to create the splint where the tests are performed. The same process could be applied to other anatomical regions [26].

### 2.1. 3D Model and Design

As reported in other previous studies, the first step was to get the 3D model of the injured part [7]. From that point, the splint is modeled over this part, considering the areas where the sensors are placed. A commercial scanner (Sense™ 3D scanner, 3D Systems Inc., Rock Hill, SC, USA) was used for the digitalization of the arm at a distance of 40 cm according to the manufacturer’s instructions. The technical specifications of the scanner are shown in Table 1.

**Figure 1:**
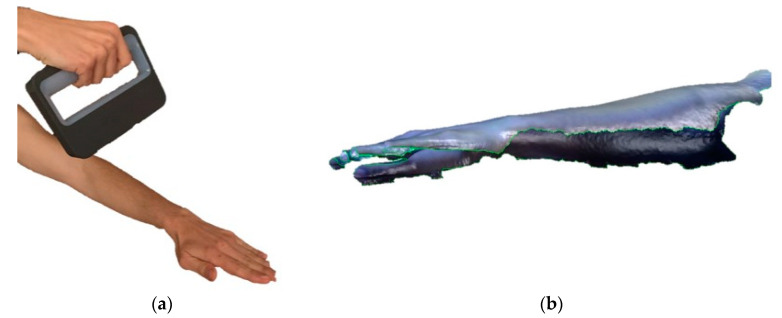
Scanning process of the arm to get the digital model (**a**) and points cloud (**b**).

It is possible to scan the entire area, at once or in several times if necessary due to limitations produced by a possible injury. Later, the software joins the points cloud. Figure 1 shows the scan process, and the 3D point cloud obtained the total time for the scanning process is 6 min.

**Figure 2:**
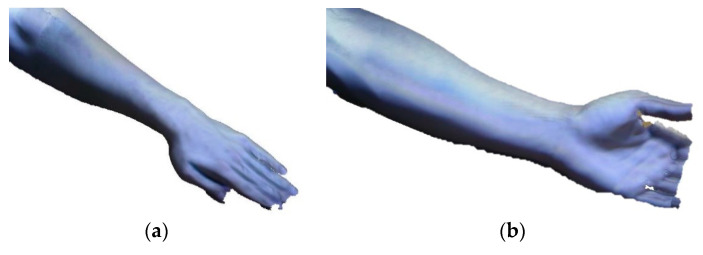
3D model of the arm obtained after the scanning process: top side (**a**) and bottom side (**b**).

Once the 3D point cloud is obtained, post-processing is carried out by using a 3D CAD software Geomagic FreeForm (3D Systems, Inc., United States). The 3D scanned models are shown in Figure 2.

**Figure 3:**
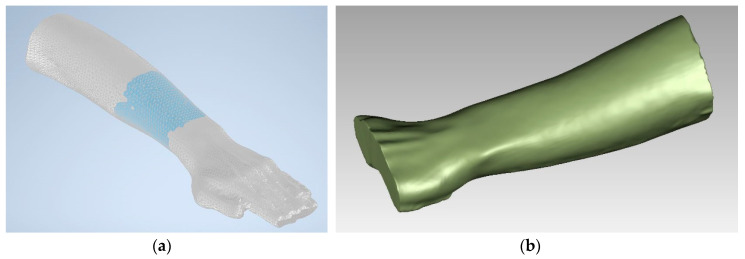
Post processed: mesh of the scanned arm (**a**) and solid digital model obtained from the clean mesh (**b**).

With this software, it was possible to clean the point cloud by filling the holes due to noise appearing during the scanning process and by correcting with a smoothing algorithm any minor anomalies due to some misplaced points of the cloud or due to surfacing process. With this procedure, it was possible to obtain a simpler mesh of the arm by limiting the area of the interest started from a point cloud. Starting from this mesh and creating an offset of 0.5 mm, the solid which is the basis of the splint is created. Figure 3 shows the mesh of the scanned arm and the solid digital model obtained.

**Figure 4:**
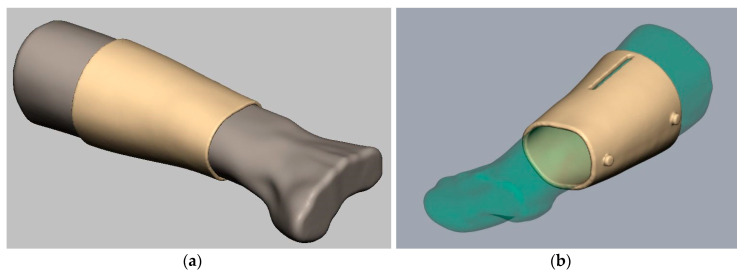
Initial solid obtained from the points cloud (**a**) and initial stage of the design, including the hole for the temperature sensor and closing buttons (**b**).

The solid digital model shown in Figure 3b represents the first step of the design of the splint. In fact, the solid surface created represents the internal face of the splint in direct contact with the skin of the patient’s arm. Once this reference surface of the splint has been created, a 5 mm thickness solid is generated as shown in Figure 4.

**Figure 5:**
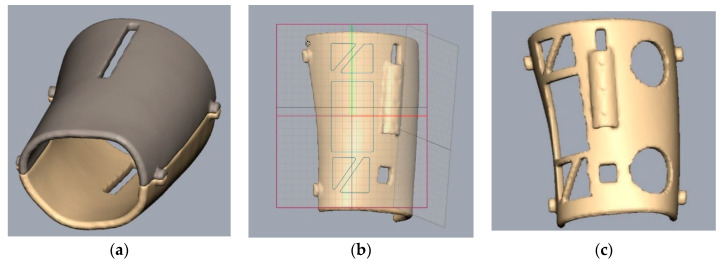
Different steps during the modeling of the splint: division of the splint (**a**), design of different sensor holes (**b**), and final top side splint (**c**).

**Figure 6:**
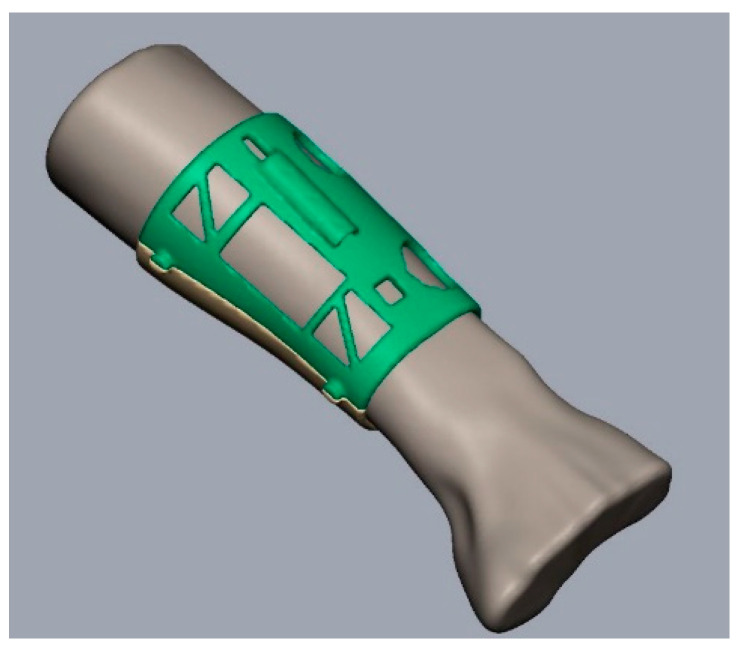
Final result of the modeled splint over the patient arm.

Once the body of the splint has been created, it was necessary to divide it in two parts to make the mounting possible, which is later joined with a mechanical closure (Figure 5a). In addition, the places for the sensors are designed and some holes are introduced as shown in the Figure 5a–c. The function of these holes and windows is manifold. In fact, they allow, on the one hand, direct medical inspection of the injured part, aeration of the skin in contact with the splint and, on the other, the possibility of using electromedical devices for rehabilitation during immobilization of the limb. Figure 6 shows the final modeled splint over the initial 3D model.

### 2.2. Sensing Technologies

In this section, devices for data acquisition in the IoT-based healthcare system and used in this study are discussed. When an injury occurs, normal inflammatory signs such as increased skin temperature, edema, changes in skin color, and pain may appear. In addition, cool skin and increased sweating are symptoms of different problems that the patient may be suffering from [27,28].

After the scanning, the splint is designed according to the 3D obtained model, incorporating the sensors housings. These sensors will be used to get the information of the splint and patient. At this point in the study, the sensors are custom positioned in the exact areas of the anatomical part to be monitored. The position adjustments and the verification of their correct behavior and setting will also be allowed.

The main parameters considered to be studied are pressure, and temperature. These aspects are combined to determine any kind of unexpected evolution of the treatment as inflammatory processes, and temperature increments due to inflammation or infections in the affected area. In addition, it will be studied the possibility of detecting changing colors of the skin and humidity. The sensors incorporated to the splint are: two temperature sensors in direct contact with the skin (DS18B20) [29], one temperature and humidity sensor to get the temperature and humidity between the skin and the splint (DHT22) [30], two pressure sensors (DF9-40) [31], and one presence sensor (CNY70) [32]. The technical specifications of all sensors are summarized in Table 2.

The DS18B20 sensors (Dallas Semiconductor™ Maxim Integrated Products, Inc., Dallas, TX, USA) are 1-Wire digital temperature sensor. It reports degrees in Celsius, with a precision of 9 to 12-bit. Each sensor has a unique 64-Bit serial number etched into it, which allows a huge number of sensors to be used on one data bus.

The DHT22 sensor (Aosong Electronics Co., Ltd., Guangzhou, China) utilizes exclusive digital-signal-collecting-technique and humidity sensing technology, its sensing elements is connected with 8-bit single-chip computer.

The DF9-40 sensor (Zhengzhou Winsen Electronics Technology Co., Ltd., Zhengzhou, China) is a film pressure based on flexible pressure sensing technology. In order to measure the applied force with an Arduino, it will be necessary to build a voltage divider circuit with a pull-down resistor. This circuit creates a variable voltage output that can be read by the ADC (analog to digital converter) input of the microcontroller. It means that for a simple force-to-voltage conversion, the force sensing resistor (R_*FSR*_) device is tied to a measuring resistor (R_*M*_) in a voltage divider. In this case, a 10kΩ resistor will be used. The output voltage (V_*out*_) which is possible to measure with the Arduino is described by the following equation:

(1) $$V_{out}=\frac{V_{cc}\cdot \;R_{M}}{R_{M}+R_{FSR}}.$$

Note that the output voltage measure is the voltage drop across the pull-down resistor. When no force is applied, the FSR resistance will be really high, for example, 10 MΩ. Using a 10 kΩ pull-down resistor and a V*_CC_* of 5 V, results in the following output when no force is applied V*_OUT_* = 0.005 V. When the FSR is pressed, the resistance goes down to roughly 200 Ω. This results in the following output voltage V*_OUT_* = 4.9. Similar developments have been carried out in other studies for other medical applications [19].

The CNY70 sensor (Vishay Telefunken, Heil- bronn, Germany) is a reflective sensor that includes an infrared emitter and phototransistor in a leaded package with a daylight blocking filter. This sensor can detect the quantity of light reflected on a surface depending on the distance. However, if we set a distance, it is possible to detect different colors depending on the infra-red light absorbed by that color. It will be tested if it is possible to detect color changes on the skin. In order to use this sensor, is necessary to build a voltage divider circuit with a pull-down resistor as in case of the pressure sensor. In this case, a 39 kΩ is used.

All these sensors have been controlled by using an Arduino UNO board [33], which will get info from the four sensors and combine to achieve a full diagnostic.

### 2.3. Additive Manufacturing

**Figure 7:**
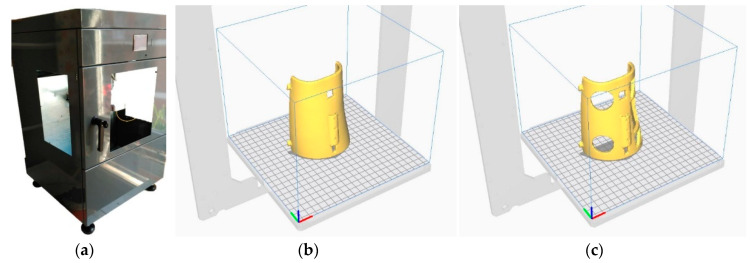
TotalPrinter machine developed to work with biomaterials (**a**). G-Code process generation for 3D printing for the different parts of the splint (**b**) and (**c**).

After the digital modeling and design process, the file obtained is sent to a slicer that prepares the part to be produced by using the additive manufacturing process (Figure 7). The splint was manufactured by using a fusion deposition modeling (FDM) 3D printer “TotalPrinter” machine (Figure 7a). This 3D printer was designed, developed, and manufactured in the Additive Manufacturing and Rapid Prototype Laboratory of the Escuela Técnica Superior de Ingeniería y Diseño Industrial at Universidad Politécnica de Madrid. This 3D printer allows continuous temperature and humidity control of the printing chamber and of extruder temperature with the aim of optimizing the process [34] and obtaining an homogeneous splint in terms of adhesion of the various printing layers, density of the material, and mechanical characteristics [35]. In addition, this 3D printing machine allows continuous monitoring of the thickness of the PLA filament used as reported in the study of Soriano et al. (2018) [36] and in the study of J. M. D. A. Del Burgo et al. (2018) [37].

The material chosen for the prototype was biocompatible material PLA filament with a diameter of 1.75 mm. The vertical printing position was chosen for the two parts of the splint as shown in the Figure 7b,c by taking in account the volume of the printing chamber (200 mm × 200 mm × 400 mm). The printing parameters used for the manufacturing of the arm splint are shown in the Table 3. These parameters were chosen according to the material manufacturer’s recommendations to obtain a sufficiently rigid and quality structure. 

## 3. Results 

In this section, the following is presented: the splint assembled with the sensors placed in their housings, the configuration of the micro controller for the collection of the monitored parameters, and the results obtained in the sampling.

### 3.1. 3D Model and Design

**Figure 8:**
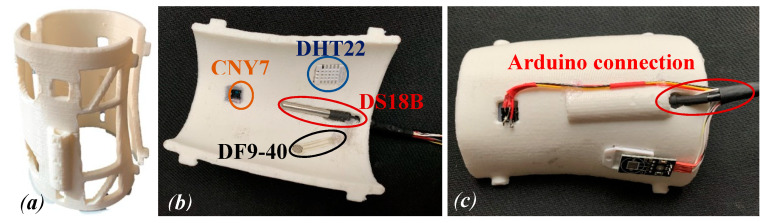
The real model of the arm splint obtained from the 3D printer (**a**). Splint assembled with the different sensors (**b**) and wires for the connection to the Arduino board (**c**).

Figure 8a shows the two parts of the arm splint prototype. Figure 8a,b shows one part of the arm splint with the sensors and wires, for the connection to the Arduino, fully integrated.

**Figure 9:**
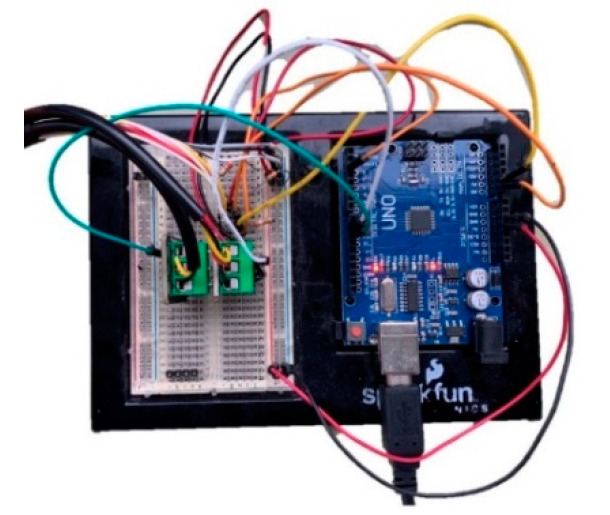
Electronic board wiring to get the data during the tests.

Once the model is prepared, it is necessary to program the Arduino board to get the data from these sensors, and to design a small circuit that includes the necessary electronic parts to connect the sensors. For this study, the data are obtained directly from the serial communication between the Arduino board and the PC. Figure 9 shows the developed electronic adopted in this study. However, these data could be sent easily to a Bluetooth or Wi-Fi device like a smartphone or tablet. It is important to report that the two contact temperature sensors and the two pressure sensors are treated digitally to get just one resultant contact temperature and pressure.

### 3.2. Sensing Technologies

**Figure 10:**
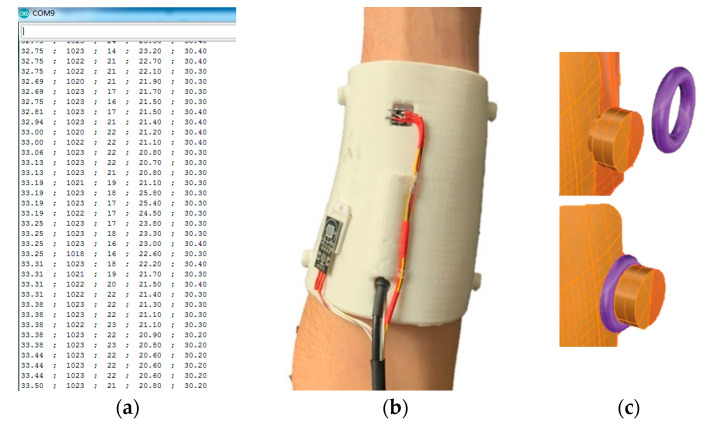
Example of the serial data collection using the Arduino board (**a**), real splint prototype with electrical wiring mounted over the arm (**b**) and detailed view of the closing buttons (**c**).

In order to test the arm splint assembled with the sensors connected to the Arduino board, the splint was fixed to the originally scanned arm for 1.5 h. The data from the sensors were collected using the Arduino serial monitor every 3 s and sent to excel to plot some graphs. Figure 10 shows the serial monitor of the acquired data while the splint was fixed to the arm of the volunteer. The obtained data are shown and explained in the following sections.

#### 3.2.1. Temperatures

**Figure 11:**
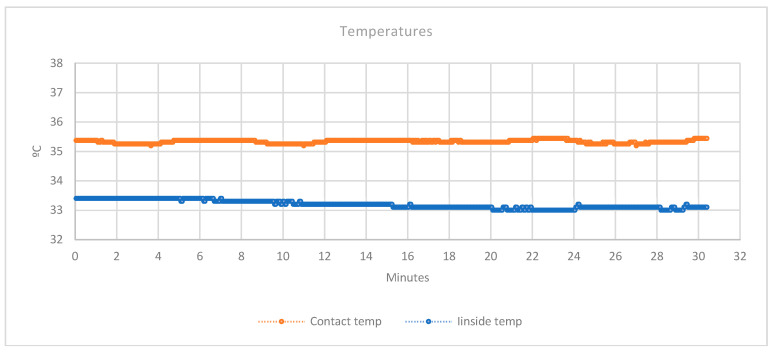
Temperatures graph.

In Figure 11, the mean temperature of the two sensors in contact with the skin is shown in orange. The temperature inside the splint is shown in blue.

It is possible to see that the sensors in contact with the skin, registered a temperature slightly lower than the normal temperature of a person (36 °C). This fact, is produced because part of the surface of the sensors, is not in real contact with the skin. Due to this, it appears a gradient of temperature between the body and the environment. However, the distance between the skin and the surface of the sensor that is not in contact with the skin, is around 4 mm. This distance is not enough to interfere with the correct sensor operation. What the authors are looking for in this study with these sensors, is to detect an increment or decrement of the temperature, compared with the historical. For this reason, the surface of the sensor not in contact will not interfere to detect correctly the variations.

As it is possible to see in the graph, the second sensor (in blue), that is completely separated from the skin, registered around two degrees less than the sensors in contact. With just these readings, a variation of the temperature is perfectly detected, which could be a symptom of inflammation or infection of the area. This info may be used with the humidity levels to discover anomalies or different situations during the treatments.

#### 3.2.2. Humidity

**Figure 12:**
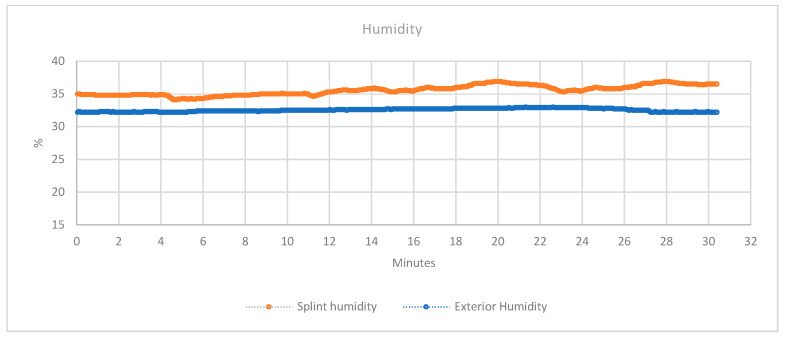
H Collected data of the humidity sensor.

The knowledge of this parameter is crucial to avoid the appearance of bacteria due to humidity, especially in the case of surgery or injuries. In addition, as explained previously, cool skin and increased sweating are symptoms of some problems that may appear after an injury [27]. Figure 12 shows the percentage of humidity registered during the test. The orange line shows the humidity between the splint and the skin. The blue line shows the environmental humidity (which was sampled with a second similar sensor). These data change due to the environmental conditions and the humidity generated by the patient through sweat.

#### 3.2.3. Pressure

**Figure 13:**
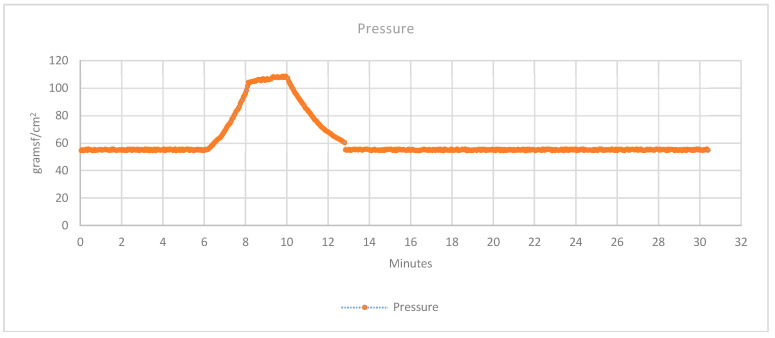
Collected data of the pressure sensor 3.2.4 Presence.

The used sensor has a surface of 1 cm^2^. Figure 13 shows the mean pressure detected by the two sensors. It is possible to see in the graph that at normal state, a pressure of 60 gf/cm^2^ is detected. This pressure is produced by the normal pressure of the splint over the body so that the position of the joint is maintained for the treatment. For the tests, between min 6 and min 12, a force over the splint is applied, by simulating an inflammatory process. This variation is detected by the sensors, reading a pressure change from 60 to 100 gf/cm^2^. The graph shows the actual pressure and also the historical. With this information, a specialist may check if the pressure is higher or lower than the previous days, in order to know if the area is suffering an inflammatory process. The doctor may check the data as often as necessary. Thereby, when the pressure is increased due to the inflammation of the area, the sensors perfectly detect the increment of the mass inside the splint.

**Figure 14:**
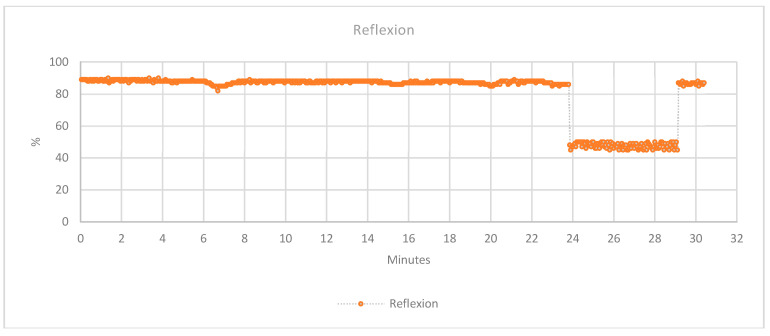
Collected data of the light reflection sensor over the skin.

The main idea of the presence sensor is to detect, whereas the splint is fixed correctly. Moreover, it is a sensor that detects the light reflection, so it was pretended to be used to detect color change of the skin. However, it was not possible to use the sensor for this propose in this study. The colors were not correctly detected with enough sensibility to detect a redness of the skin due to inflammation or other problems. In fact, it should be remembered that in this study, the patient, on whom the splint has been tested, is not affected by any pathology that can cause redness or, at least, the change of skin color. Figure 14 shows the percentage of the light reflected over the skin. As it is possible to see in the graph, it is perfectly detected, whereas the splint is not fixed correctly between the min 24 and 29. In fact, in this temporal space simulated in not perfect assembly and adhesion of the splint to the patient’s arm.

## 4. Discussions

For nearly half a century, research has been conducted on humans [38] and animals [39,40], showing the correlation between temperature patterns and medical conditions. The temperature variation of an area of the body is produced by cell metabolism and local blood flow. The increments in temperature are normally the result of an increase of these factors, although it is true that blood flow plays the most important role. Moreover, with some disease processes or during the different bone formation stages and fractures repair, it may occur a reduction in blood flow to the affected areas [41]. This will also produce some alterations in surface temperature.

In the case of using a conventional arm splint, the weight of the material may produce pain in the neck. In addition, the lack of ventilation and the difficulty of bathing could produce allergies and itching due to the poor hygiene of the skin and the device. Another negative point of traditional cast implants is the difficulty of handling it in some daily activities by patients [7].

This study proposes the implementation of a series of sensors to detect possible problems during the whole time of the treatment. The duration of the treatment does not interfere with the data collection and interpretation. In fact, the collection may be carried out periodically, depending on the specialized doctor necessities and on the injury evolution. For this reason, the data collection is carried out as a sign that the system works.

As it is possible to see in the charts obtained from the sensors, it is perfectly possible to detect temperature variations, pressure increment, and humidity inside the splint. Besides, if more temperature sensors are situated in the splint, it would allow to create a digital mapping of the different areas under the splint. This information to quantify temperature changes in skin surface indicates both hot and cold responses which may co-exist if the pain associated with an inflammatory focus excites an increase in sympathetic activity [27]. Continuous monitoring of these parameters allows to know the progression of area at any time of the treatment. This allows early detection of possible vascular, muscular, or joint complications that are difficult to observe without the removal of the immobilization splint or through severe symptoms caused by often irreparable damage.

The temperature registered by the sensor in contact with the skin, is slightly lower than the normal temperature. This is due to the temperature gradient that occurs on the sensor surface. The sensor surface in contact with the skin is about a 40%. The rest of the surface is covered by the splint but not in contact with the skin. This fact produces a difference of temperature between different points of the sensor. The metallic surface tends to get an equilibrium due to thermal conductivity [38]. The sensor gets a final temperature that is not the real skin temperature. However, this fact does not interfere with the main purpose of this sensor, which is to detect incremental temperature changes.

Temperature, humidity, and pressure are correctly detected by the sensors. In addition, the presence sensor detects perfectly, whereas the splint is correctly fixed. However, the presence sensor cannot be used for its second propose, detecting color changes by the variations of the reflection due to the skin color. This sensor has not enough sensibility to detect light variations. Moreover, it does not include an RGB sensor to detect different wavelengths, making it really difficult to use the sensor for this purpose. Therefore, the use of such a sensor for the proposed purpose is not estimated.

## 5. Conclusions

The main objective of this work was the study, design, and manufacture of a smart and immobilization splint to monitor different parameters about the real evolution of an injury. This makes it possible to have real-time data to diagnose any kind of problem that is not possible to detect with traditional splints. The current state of the technology allows the realization of particular and individualized immobilization splints by using advanced manufacturing, based on fused deposition modeling, 3D digitalization, and reverse engineering. Moreover, the knowledge of these parameters, might reduce the curing time as some treatments can be applied in this kind of splint but not in traditional splints.

In this study it is shown an interesting and really possible evolution of the traditional splints, to provide them with the term “intelligent”. The great possibilities that it offers by this kind of splint is fully demonstrated. However, there are some improvements that are proposed to be carried out in future studies:

- Implementation of a sensor to detect color changes of the skin: This would make it possible to detect bruising and redness, which would allow to get more data for the diagnostic.
- Sending these data to a Bluetooth device that allows remote medical monitoring of the treatment applied and the application of an alarm system in the event of complications, as well as the generation of the data dump in a database for study.
- Integration into functional splints based on rehabilitation techniques in the immobilization phase. This would make it necessary to incorporate a battery to get a full autonomous system.

To finish this study, the main objective is considered to have been achieved, by the incorporation of these sensors and demonstrated that it is possible to detect complications in the treatment of injuries using immobilization splints.

## Figures and Tables

**Table 1:** Technical specifications of the Sense™ 3D scanner.

Maximum Scan Volume	2 × 2 × 2 (m)
Minimum Scan Volume	0.2 × 0.2 × 0.2 (m)
Working Distance	0.2–1.6 (m)
Number of Cameras	2
Class Certified Laser Product	1
Resolution at 0.5 m	1 (mm)

**Table 2:** Technical specifications of the sensor.

Sensor Serial Number	DS18B20	DHT22	DF9-40	CNY70
Dimensions (mm)	6 × 6 × 50	15 × 7.7 × 20	40 × 20 × 0.25	7 × 7 × 6
Power voltage (*V*)	3.0–5.5	3.3–6	5	5
Working range	−55 °C to 125 °C	−40 °C to 80 °C 0 to 100% RH	0–500 g	0 to 10 mm
Resolution	± 0.0625 °C	0.1 °C 0.1% RH	14.5 g	−

**Table 3:** Printing parameters.

Layer height [mm]	0.2
Extruder [mm]	0.4
Print density [%]	40
Thickness perimeter of closure of each layer [mm]	1
Print speed [mm/s]	60
Temperature [°C]	210

## References

[1] Jammalamadaka U., Tappa K. (2018). Recent advances in biomaterials for 3D printing and tissue engineering. J. Funct. Biomater..

[2] Tappa K., Jammalamadaka U. (2018). Novel biomaterials used in medical 3D printing techniques. J. Funct. Biomater..

[3] Bandyopadhyay A., Bose S., Das S. (2015). 3D printing of biomaterials. MRS Bull..

[4] Chia H.N., Wu B.M. (2015). Recent advances in 3D printing of biomaterials. J. Biol. Eng..

[5] Guvendiren M., Molde J., Soares R.M.D., Kohn J. (2016). Designing Biomaterials for 3D Printing. ACS Biomater. Sci. Eng..

[6] Valášek P., D’Amato R., Müller M., Ruggiero A. (2018). Musa textilis Cellulose Fibres in Biocomposites—An Investigation of Mechanical Properties and Microstructure. BioResources.

[7] Blaya F., Pedro P.S., Silva J.L., D’Amato R., Heras E.S., Juanes J.A. (2018). Design of an Orthopedic Product by Using Additive Manufacturing Technology: The Arm Splint. J. Med. Syst..

[8] Affatato S., Ruggiero A., De Mattia J.S., Taddei P. (2016). Does metal transfer affect the tribological behaviour of femoral heads? Roughness and phase transformation analyses on retrieved zirconia and Biolox® Delta composites. Compos. Part B Eng..

[9] Lozano M.T.U., D’Amato R., Ruggiero A., Manzoor S., Haro F.B., Méndez J.A.J. A study evaluating the level of satisfaction of the students of health sciences about the use of 3D printed bone models. Proceedings of the Sixth International Conference on Technological Ecosystems for Enhancing Multiculturality-TEEM’18.

[10] Ugidos Lozano M.T., Blaya Haro F., Ruggiero A., Manzoor S., Nuere Menendez-Pidal S., Juanes Méndez J.A. (2018). Different Digitalization Techniques for 3D Printing of Anatomical Pieces. J. Med. Syst..

[11] Ghai S., Sharma Y., Jain N., Satpathy M., Pillai A.K. (2018). Use of 3-D printing technologies in craniomaxillofacial surgery: A review. Oral Maxillofac. Surg..

[12] García N.M., Blaya F., Urquijo E.L., Heras E.S., D’Amato R. (2019). Oral appliance for Obstructive Sleep Apnea: Prototyping and Optimization of the Mandibular Protrusion Device. J. Med. Syst..

[13] Montesdeoca N., Lechosa E., Haro F.B., D’Amato R., Juanes J.A. (2019). Design of thermoplastic oral appliance with mouth opening control to treat obstructive sleep apnea. Proceedings of the ACM International Conference Proceeding Series.

[14] Mulford J.S., Babazadeh S., Mackay N. (2016). Three-dimensional printing in orthopaedic surgery: Review of current and future applications. ANZ J. Surg..

[15] Lunsford C., Grindle G., Salatin B., Dicianno B.E. (2016). Innovations With 3-Dimensional Printing in Physical Medicine and Rehabilitation: A Review of the Literature. PM R.

[16] Fitzpatrick A.P. (2017). Design of a Patient Specific, 3D printed Arm Cast. KnE Eng..

[17] Cernohorsky J., Cadek M. (2017). Smart rehabilitation splint. Advances in Mechanism Design II.

[18] Evill J., Evill O. Cortex Evill. https://www.evilldesign.com/cortex.

[19] Robin O., Claude A., Gehin C., Massot B., McAdams E. (2020). Recording of bruxism events in sleeping humans at home with a smart instrumented splint. J. Craniomandib. Sleep Pract..

[20] Goncu-Berk G., Topcuoglu N. (2017). A Healthcare Wearable for Chronic Pain Management. Design of a Smart Glove for Rheumatoid Arthritis. Des. J..

[21] Chiu Y.H., Chen T.W., Chen Y.J., Su C.I., Hwang K.S., Ho W.H. (2018). Fuzzy logic-based mobile computing system for hand rehabilitation after neurological injury. Technol. Health Care.

[22] Dimitrov D.V. (2016). Medical internet of things and big data in healthcare. Healthc. Inf. Res..

[23] Yin Y., Zeng Y., Chen X., Fan Y. (2016). The internet of things in healthcare: An overview. J. Ind. Inf. Integr..

[24] Zanella A., Bui N., Castellani A., Vangelista L., Zorzi M. (2014). Internet of things for smart cities. IEEE Internet Things J..

[25] Fan Y.J., Yin Y.H., Xu L.D., Zeng Y., Wu F. (2014). IoT-based smart rehabilitation system. IEEE Trans. Ind. Inform..

[26] Blaya F., Pedro P.S., Pedro A.B.S., Lopez-Silva J., Juanes J.A., D’Amato R. (2019). Design of a Functional Splint for Rehabilitation of Achilles Tendon Injury Using Advanced Manufacturing (AM) Techniques. Implementation Study. J. Med. Syst..

[27] Ju X., Nebel J.-C., Siebert J.P., Gong H., Cai Y., Chatard J.-P. (2005). 3D thermography imaging standardization technique for inflammation diagnosis. Proceedings of the Infrared Components and Their Applications.

[28] Schlereth T., Drummond P.D., Birklein F. (2014). Inflammation in CRPS: Role of the sympathetic supply. Auton. Neurosci. Basic Clin..

[29] Dallas Semiconductor (2002). Programmable Resolution 1-Wire® Digital Thermometer.

[30] Liu T. (2015). Digital-Output Relative Humidity & Temperature Sensor/Module DHT22 (DHT22 Also Named as AM2302).

[31] Datasheet Film Pressure Sensor DF9-40@10kg V2.0. https://www.winsen-sensor.com/d/files/df9-40%4010kg.pdf.

[32] Reflective Optical Sensor with Transistor Output. https://www.tme.eu/Document/5c845fc67f29d9b4e8f31810ed773b0f/cny70.pdf.

[33] Arduino Arduino Uno Rev3|Arduino Official Store. https://store.arduino.cc/arduino-uno-rev3.

[34] Haro F.B., de Agustín del Burgo J.M., D’Amato R., Islán M., Heras E.S., Alonso J.M.G., Mendez J.A.J. (2019). Monitoring an Analysis of Perturbations in Fusion Deposition Modelling (FDM) Processes for the Use of Biomaterials. J. Med. Syst..

[35] Haro F.B., de Agustín del Burgo J.M., D’Amato R., Marcos M.I., Heras E., Alonso J.M.G. (2018). Monitoring of the additive manufacturing process for the use of biomaterials in medical field. Proceedings of the Sixth International Conference on Technological Ecosystems for Enhancing Multiculturality-TEEM’18.

[36] Soriano Heras E., Blaya Haro F., de Agustín del Burgo J.M., Islán Marcos M., D’Amato R. (2018). Filament advance detection sensor for fused deposition modelling 3D printers. Sensors.

[37] Del Burgo J.M.D.A., D’Amato R., Méndez J.A.J., Ramírez A.S., Haro F.B., Heras E.S. (2019). Real time analysis of the filament for FDM 3D printers. ACM International Conference Proceeding Series.

[38] Lawson R. (1956). Implications of surface temperatures in the diagnosis of breast cancer. Can. Med. Assoc. J..

[39] Ströberg B. (1974). The Use of Thermography in Equine Orthopedics. Vet. Radiol..

[40] Wang L., Guo T.Z., Wei T., Li W.W., Shi X., Clark J.D., Kingery W.S. (2016). Bisphosphonates Inhibit Pain, Bone Loss, and Inflammation in a Rat Tibia Fracture Model of Complex Regional Pain Syndrome. Anesthesia Analg..

[41] Rundle C.H., Wang H., Yu H., Chadwick R.B., Davis E.I., Wergedal J.E., Lau K.H.W., Mohan S., Ryaby J.T., Baylink D.J. (2006). Microarray analysis of gene expression during the inflammation and endochondral bone formation stages of rat femur fracture repair. Bone.

